# Short-Term Effects of Anodal Transcranial Direct Current Stimulation on Endurance and Maximal Force Production: A Systematic Review and Meta-Analysis

**DOI:** 10.3390/jcm8040536

**Published:** 2019-04-18

**Authors:** Carlos Alix-Fages, Salvador Romero-Arenas, Marcos Castro-Alonso, David Colomer-Poveda, Dan Río-Rodriguez, Agustín Jerez-Martínez, Miguel Fernandez-del-Olmo, Gonzalo Márquez

**Affiliations:** 1 Department of Physical Education and Sport, Faculty of Sport Sciences, Catholic University of Murcia (UCAM), 30107 Murcia, Spain; calix@alu.ucam.edu (C.A.-F.); sromero@ucam.edu (S.R.-A.); dcolomer@ucam.edu (D.C.-P.); ajerez@alu.ucam.edu (A.J.-M.); 2 Department of Physical Education, University of A Coruña, 15279 La Coruña, Spain; marcoscastroalonso@gmail.com (M.C.-A.); ganceus@gmail.com (D.R.-R.); mafo73@gmail.com (M.F.-d.-O.); 3 Department of Education, King Juan Carlos University, 28933 Madrid, Spain

**Keywords:** Non-invasive brain stimulation, time to task failure, maximal voluntary contraction, primary motor cortex, prefrontal cortex

## Abstract

The purpose of the present systematic review and meta-analysis was to explore the effects of transcranial direct current stimulation (tDCS) on endurance (i.e., time to task failure (TTF)) and maximal voluntary contraction (MVC). Furthermore, we aimed to analyze whether the duration of stimulation, the brain region targeted for stimulation, and the task performed could also influence motor performance. We performed a systematic literature review in the databases MEDLINE and Web of Science. The short-term effects of anodal tDCS and sham stimulation (placebo) were considered as experimental and control conditions, respectively. A total of 31 interventions were included (MVC = 13; TTF = 18). Analysis of the strength-related tDCS studies showed small improvements in the MVC (SMD = 0.19; 95% CI = −0.02, 0.41; *p* = 0.08). However, the results of the endurance-related interventions indicated a moderate effect on TTF performance (SMD = 0.26; 95% CI = 0.07, 0.45; *p* = 0.008). Furthermore, the sub-analysis showed that anodal tDCS over M1 and stimulation durations longer than 10 min produced the best results in terms of TTF performance enhancement. Additionally, the effects of anodal tDCS were larger during full body exercises (i.e., cycling) when compared to uniarticular tasks. In conclusion, the current meta-analysis indicated that anodal tDCS leads to small and moderate effects on MVC and TTF, respectively.

## 1. Introduction

Non-invasive brain stimulation paradigms have been receiving increased interest in recent years as tools for modulating cortical excitability and behaviour in a range of clinical settings and experimental conditions. Transcranial direct current stimulation (tDCS) is a form of stimulation that holds particular promise in both of these settings as it is non-invasive, painless, well-tolerated [1] and safe [2]. This form of stimulation consists of delivering a constant and weak electrical current to the brain by placing two or more electrodes over the scalp [3]. Stimulation lasting for longer than nine minutes might induce significant after-effects on cortical excitability that could last up to 90 min [4,5]. These effects are mainly due to changes in resting membrane potential of the targeted cells [1]. However, the effects of tDCS on cortical excitability are polarity specific, since anodal tDCS increases cortical excitability while cathodal tDCS produces the opposite effect [3].

This neuromodulatory technique has been widely used for the treatment of different neurological [6,7] and psychiatric disorders/conditions [8,9], as well as to improve learning [10] and memory [11]. Recently, tDCS has been also tested as a tool to boost important aspects of athletic/motor performance such as strength [12,13,14] and endurance [13,15,16,17]. A recent scoping mini-review [18] suggests that the mechanisms underlining the additive effects produced by anodal tDCS on motor performance could be related to an increased cortical excitability within the M1 (Primary Motor Cortex) which in turn led to reductions in supraspinal fatigue [15,17] and ratings of perceived exertion [19,20]. 

However, the effects of anodal tDCS on the maximal voluntary strength are inconclusive. Some studies found a positive effect of anodal stimulation on maximal isometric voluntary contraction [12,14,21,22] or an increased mechanical power output during a vertical jump [23]. Conversely, other studies did not find any effect of anodal tDCS in maximal strength capabilities in healthy subjects [13,24,25]. Regarding the effects of anodal tDCS on endurance performance (i.e., time to task failure -TTF-), most of the literature [15,16,17,19,20,26,27,28,29,30] have found a positive effect over the time to task failure (TTF) tests, although, some studies reported no effects [13,24,31,32,33]. 

Such inconsistencies might be related to different factors such as (i) the characteristics of the stimulation protocol; (ii) the brain region under stimulation and (iii) the task under evaluation.

Duration of stimulation could be an important determinant of the stimulation after-effects. Nitsche and Paulus [4] revealed sustained elevations of cortical excitability after thirteen minutes anodal tDCS (up to 90 min) compared to shorter stimulation periods (5–7 min), which lasted for no longer than five minutes. Therefore, it seems that this may also influence behavioural results. In this regard, Williams et al. [17] have found that during a submaximal isolated isometric TTF test the group that received anodal tDCS during the entire test significantly improved the TTF, but the group that was stimulated only during the 50% of the TTF test did not. The stimulation electrodes montage also seems to influence tDCS effects, with extracephalic montages leading to higher TTF duration in comparison to cephalic montages [27]. 

Most of the studies targeted M1 given its prominent role in the regulation of exercise capacity (e.g., descending neural drive and development of supraspinal fatigue; [34]). Those studies have found both positive and negative effects of tDCS on TTF [15,16,17,20,26,29,30] and maximal voluntary strength [12,14,20,21,22]. Results were also inconclusive when the tDCS was applied over dorsolateral prefrontal cortex (DLPFC) or insular cortex (IC), with positive [19,28] or no effects [16,32] on motor performance. In this regard, Okano et al. [19] observed that anodal, but not sham, tDCS over IC modulated autonomic response (i.e., heart rate variability) during an incremental test to exhaustion that leads to higher power output at the end of the test. Angius et al. [20] demonstrated that 10 min anodal tDCS induced an increase in M1 excitability that was then associated with higher performance during a TTF test. Therefore, it seems that the stimulation of different brain regions may lead to similar behavioural results (i.e., increased TTF).

Finally, the task performed (i.e., single joint exercise versus whole-body dynamic exercise) to study the effects of anodal tDCS on motor performance is of relevance, since metabolic, cardiorespiratory, and psychological demands are completely different when comparing single joint and whole-body exercises, and therefore it could differentially affect brain activity [18]. Although, single joint exercises permit a more controlled examination of the physiological effects of anodal tDCS, whole-body exercise better reflect real sport situations [18], and thus, it may provide the necessary ecological validity for being used in field conditions. 

Based on above-mentioned observations, it seems rational to clarify the following questions: (1) does anodal tDCS improve TTF and maximal strength capabilities; (2) does the duration of the anodal tDCS intervention influence the effects on motor performance; (3) does the stimulated brain region differentially affect motor performance; (4) does the task influence the effect induced by anodal tDCS. Therefore, the purpose of the present systematic review and meta-analysis was to quantify any potential systematic effects that anodal tDCS may have on TTF and maximal strength capabilities. Furthermore, we also aim to analyze whether the duration of stimulation, the brain region targeted for stimulation and the task performed could also influence motor performance.

## 2. Methods

The present systematic review was performed according to the ‘Preferred Reporting Items for Systematic Review and Meta-Analysis Protocols’ (PRISMA-P) 2015 guidelines [35]. 

### 2.1. Search Strategy

A systematic literature review was conducted across the date range, January 1970 to December 2018, using the online databases MEDLINE (via PubMed), ScienceDirect and Web of Science. The search strategy was composite by two main concepts, the first one referring to non-invasive brain stimulation techniques (i.e., “tDCS” OR “a-tDCS” OR “anodal-tDCS” OR “c-tDCS” OR “cathodal-tDCS” OR “transcranial direct current stimulation”) and the other one referring to the main performance outcomes focus of this review (i.e., “strength” OR “maximal voluntary contraction” OR “MVC” OR “one repetition maximum” OR “1RM” OR “endurance” OR “time to task failure” OR “resistance” OR “time limit” OR “time to exhaustion”). The literature search was conducted by C.A.-F. The authors of published papers were contacted personally if crucial data were not reported in original papers. An additional search was made looking at the references of each included article and relevant reviews to identify additional suitable studies for inclusion.

### 2.2. Eligibility Criteria and Study Selection

After removal of duplicates the remaining articles were screened manually and papers providing insufficient information in title and abstract were full-text screened. Papers were included in the review based on the PICOS approach. In this approach, P: stands for population, I: for intervention, C: for comparators, O: for main outcome, and S: for study design. Randomised controlled trials (S) with healthy young and old adults (i.e. from 18 to 85 years old) free of orthopaedic and neurological conditions (P), were included if measured the effects of acute administration of tDCS prior to, or during, endurance or strength tasks (I). The presence of a control group that receive sham stimulation was also required to exclude a possible placebo effect (C). Endurance tasks were considered as any effort in which subjects had to perform the task until they could no longer continue with the effort (i.e., time to task failure: TTF, time to exhaustion test, or incremental exercise testing) or maintain a predetermined minimal level of effort, independently of the nature of the task (cyclical efforts in ergometers, or isometric submaximal contractions) and lasting at least 75 s. Strength tasks were considered as any brief maximal effort in which the subject’s main aim was to generate a maximal amount of force in a movement independently of the nature of the contraction (i.e., isometric/dynamic maximal voluntary contraction (MVC)). Thus, the main outcomes (O) were time (in seconds), in the case of endurance tasks, and maximal voluntary contraction force (in N, N·m, N·Kg^−1^), in the case of strength tasks. The final inclusion/exclusion decision was made by two independent researchers (C.A.-F and G.M.).

### 2.3. Coding

Each study was coded for the following variables: authors, publication date, sample size and participants’ characteristics (i.e. age and sex), type and polarity of stimulation, stimulation and reference electrode location, duration of stimulation, current density, task performed, and primary key outcome (measures of endurance or strength performance). When some data was missed, we first contacted the authors of the original papers and if data was not facilitated, we estimated means and standard deviations from the published figures using WebPlotDigitizer software (v4.2, San Francisco, CA, USA) (https://apps.automeris.io/wpd/). 

### 2.4. Assessment of Methodological Quality

To quantify the methodological quality of the included studies, we used the Physiotherapy Evidence Database (PEDro) scale (http://www.pedro.org.au). This scale consists of 10 criteria that rates the interval validity and the presence of statistically replicable information, of which the first is not included in the total score. Each criterion is rated “yes” or “no”, with “yes” only awarded when a criterion is clearly satisfied. The maximum score that can be given is 10 if all criteria are satisfied. The cut-off score for rate a study as high quality is ≥6/10, with lower scores considered as low methodological quality. Two researchers (C.A.-F. and G.M.) rated the methodological quality of each study independently. When there was a discrepancy this was resolved by discussion until consensus was reached. The raters were not blinded to the study authors’ place of publications and results.

### 2.5. Statistical Analysis

The meta-analysis and statistical analyses were performed using Review Manager software (RevMan 5.3.5; Cochrane Collaboration, Oxford, UK) and Comprehensive Meta-analysis software (version 3; Biostat, Englewood, NJ, USA). In each study, the size of the effect of the intervention was also calculated by the difference in performance after the intervention between experimental and control (i.e., sham) conditions. Each mean difference was weighted according to the inverse variance method. Since TTF and maximal voluntary force were assessed by different methods, the mean differences were standardized by dividing them by the within-group standard deviation. The standardized mean difference (SMD) values in each trial were pooled with a random effects model. According to Cohen guidelines [36], SMD values of 0.2, 0.5, and 0.8 represent small, moderate and large effect sizes, respectively.

Heterogeneity between studies was assessed using *I*^2^ statistics. Potential moderating factors were evaluated by subgroup analysis comparing trials grouped by dichotomous variables potentially influencing performance. An arbitrary way was used as cut-off values for grouping trials. Publication bias was evaluated by estimating Begg and Mazumdar’s funnel plot asymmetry and Egger’s weighted regression test. Statistical significance was set at *p* ≤ 0.05.

## 3. Results

### 3.1. Study Selection and Characteristics

**Figure 1:**
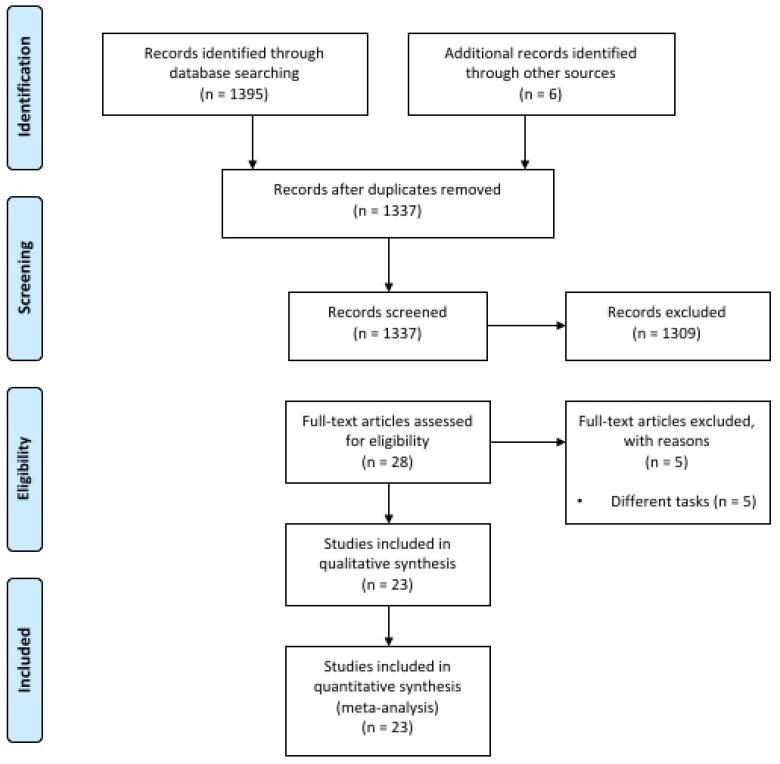
Flow diagram of the literature search.

The article-selection process resulted in the inclusion of 23 studies that resulted in 31 interventions, 13 in strength tasks [12,13,14,20,21,22,24,25,27,37,38,39] and 18 in endurance tasks [13,15,16,17,19,20,24,26,27,28,29,30,31,32,33]. The studies of Kan et al. [13], Angius et al. [20], Flood et al. [24] and Angius et al. [27] were included in both MVC and TTF analysis because they explored the effects of anodal tDCS on both strength- and endurance-related variables. Furthermore, Radel et al. [16], Williams et al. [17] and Angius et al. [27] included different tDCS interventions within the same study, which were taken separately. The flow diagram of the study selection process is depicted in Figure 1. Table 1 and Table 2 show the main characteristics of the resulting studies.

### 3.2. Study Quality Assessment and Publication Bias Evaluation

The mean quality of the studies was high. The mean score of the PEDro scale was 7.2 ± 1.0 of a possible 10 points (Table S1). As for the evaluation of potential biases, the funnel plots (Figure S1) did not indicate the presence of publication bias for the SMDs in Time to Task Failure (TTF) and maximal voluntary force in the studies included in the meta-analysis.

### 3.3. Effects of tDCS on TTF and MVC

**Figure 2:**
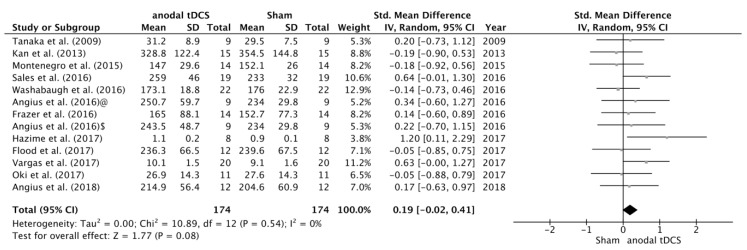
Forest plot of the comparison of MVC between anodal tDCS and sham conditions. Angius et al. [27] $—condition in which the subjects received anodal tDCS using a cephalic montage (Anode in M1 and Cathode in right DLPFC). Angius et al. [27] @—condition in which the subjects received anodal tDCS using an extracephalic montage (Anode in M1 and Cathode in shoulder).

**Figure 3:**
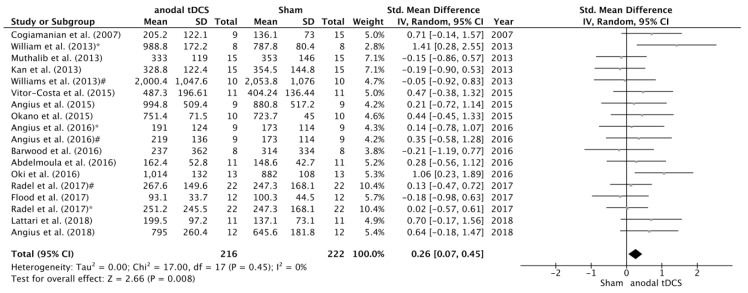
Forest plot of the comparison of TTF between anodal tDCS and sham conditions. Williams et al. [17] *—subgroup that received full time anodal tDCS during the task (i.e., submaximal isometric elbow contraction until failure). Williams et al. [17] #—subgroup that received part time anodal tDCS during the task (i.e., submaximal isometric elbow contraction until failure). Angius et al. [27] *—condition in which the subjects received anodal tDCS using a cephalic montage (Anode in M1 and Cathode in right DLPFC). Angius et al. [27] #—condition in which the subjects received anodal tDCS using an extracephalic montage (Anode in M1 and Cathode in shoulder). Radel et al. [16] *—condition in which the subjects received anodal tDCS over the PFC. Radel et al. [16] #—condition in which the subjects received anodal tDCS over the M1.

The search identified 12 studies that examined the effects of tDCS on maximal voluntary force. After data pooling, SMD between sham and anodal conditions did not reach statistical significance (SMD = 0.19; 95% CI= −0.02, 0.41; *p* = 0.08) (Figure 2). On the other hand, we have identified 18 studies that examined the effects of tDCS on TTF, and in this case the change in TTF after the intervention changed in favour the anodal versus sham condition (SMD = 0.26; 95% CI = 0.07, 0.45; *p* = 0.008) (Figure 3). No significant heterogeneity was detected in either of these analyses.

### 3.4. Subgroup Analyses of the Effects of tDCS

**Figure 4:**
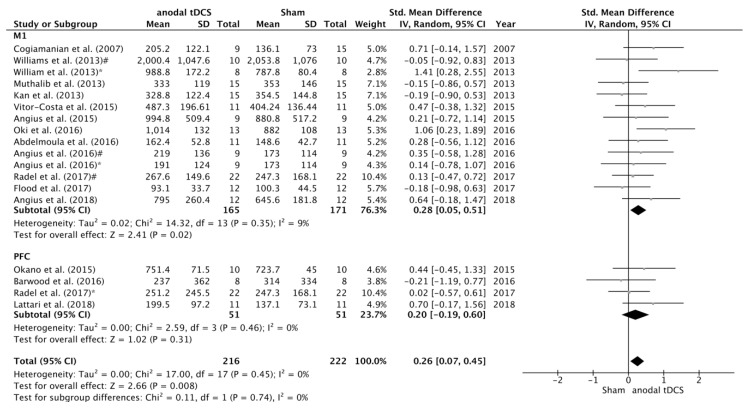
Forest plot of the subgroup analysis for the comparison of the TTF between anodal tDCS over M1 versus PFC. Williams et al. [17] *—subgroup that received full time anodal tDCS during the task (i.e., submaximal isometric elbow contraction until failure). Williams et al. [17] #—subgroup that received part time anodal tDCS during the task (i.e., submaximal isometric elbow contraction until failure). Angius et al. [27] *—condition in which the subjects received anodal tDCS using a cephalic montage (Anode in M1 and Cathode in right DLPFC). Angius et al. [27] #—condition in which the subjects received anodal tDCS using an extracephalic montage (Anode in M1 and Cathode in shoulder). Radel et al. [16] *—condition in which the subjects received anodal tDCS over the PFC. Radel et al. [16] #—condition in which the subjects received anodal tDCS over the PFC.

**Figure 5:**
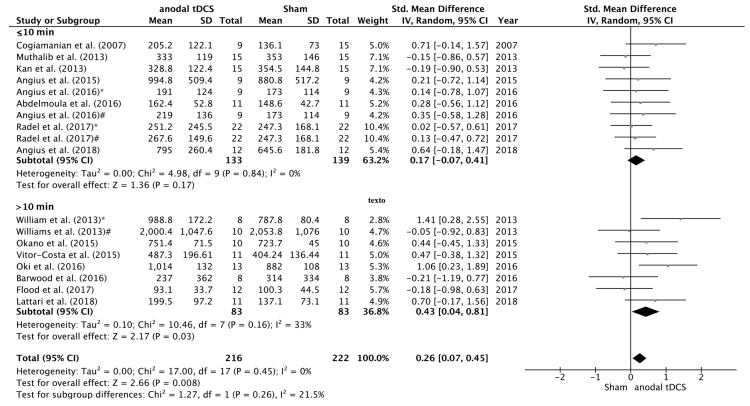
Forest plot of the subgroup analysis for the comparison of the TTF between anodal tDCS applied ≤10 min. versus >10 min. Williams et al. [17] *—subgroup that received full time anodal tDCS during the task (i.e., submaximal isometric elbow contraction until failure). Williams et al. [17] #—subgroup that received part time anodal tDCS during the task (i.e., submaximal isometric elbow contraction until failure). Angius et al. [27] *—condition in which the subjects received anodal tDCS using a cephalic montage (Anode in M1 and Cathode in right DLPFC). Angius et al. [27] #—condition in which the subjects received anodal tDCS using an extracephalic montage (Anode in M1 and Cathode in shoulder). Radel et al. [16] *—condition in which the subjects received anodal tDCS over the PFC. Radel et al. [16] #—condition in which the subjects received anodal tDCS over the PFC.

**Figure 6:**
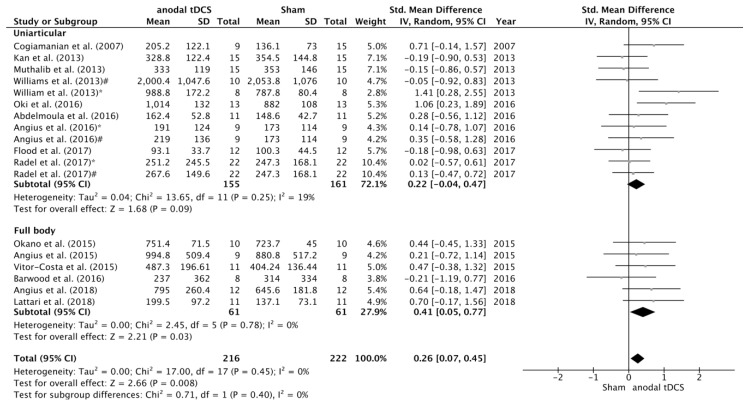
Forest plot of the subgroup analysis for the comparison of the effects of anodal tDCS on TTF between studies that used whole-body (i.e., cycling) versus uniarticular (i.e., submaximal isometric contractions until failure) tasks. Williams et al. (2013) *—subgroup that received full time anodal tDCS during the task (i.e., submaximal isometric elbow contraction until failure). Williams et al. (2013) #—subgroup that received part time anodal tDCS during the task (i.e., submaximal isometric elbow contraction until failure). Angius et al. (2016) *—condition in which the subjects received anodal tDCS using a cephalic montage (Anode in M1 and Cathode in right DLPFC). Angius et al. (2016) #—condition in which the subjects received anodal tDCS using an extracephalic montage (Anode in M1 and Cathode in shoulder). Radel et al. (2017) *—condition in which the subjects received anodal tDCS over the PFC. Radel et al. (2017) #—condition in which the subjects received anodal tDCS over the PFC.

Subgroup analyses were conducted to study the influence of potential moderating factors on the SMD between anodal and sham conditions in endurance tasks (Figure 4, Figure 5 and Figure 6). A significant increase in TTF was detected in Primary Motor Cortex (M1) subgroup on the SMD between sham and anodal conditions (SMD = 0.28; 95% CI = 0.05, 0.51; *p* = 0.02). However, there were no statistical differences on the SMD between anodal and sham conditions in the studies where the stimulation was applied over prefrontal or temporal areas (SMD = 0.20; 95% CI = −0.19, 0.60; *p* = 0.31) (Figure 4). 

Regarding duration of stimulation, the studies in which the stimulation time was >10 min the change in time to task failure after the intervention changed in favour the anodal versus sham condition (SMD = 0.43; 95% CI = 0.04, 0.81; *p* = 0.03). There were no statistical differences on the SMD between anodal and sham conditions when the stimulation time was ≤10 min (SMD = 0.17; 95% CI = −0.07, 0.41; *p* = 0.17) (Figure 5). 

With respect to task type, a significant increase in TTF was detected in full body activities subgroup on the SMD in favour of anodal versus sham condition (SMD = 0.41; 95% CI = 0.05, 0.77; *p* = 0.03), but there were no statistical differences on the SMD between experimental and control conditions in the studies using uniarticular task to failure (SMD = 0.20; 95% CI = −0.02, 0.43; *p* = 0.08) (Figure 6). There were no significant differences between complementary subgroups.

## 4. Discussion

The present meta-analysis explored the effects of a single session of anodal tDCS on athletic performance (i.e. maximal strength and muscular endurance). Analysis of the strength-related tDCS studies, comprised a total of 174 participants in 13 studies, showed a small effect on maximal voluntary force (SMD = 0.19; *p* = 0.08). However, the results of the 18 endurance-related interventions, which comprised 216 participants, indicated a moderate effect on TTF performance (SMD = 0.26, *p* = 0.008). Furthermore, the sub-analysis showed that full body (i.e., cycling) exercises, M1 stimulation and more than 10 min of stimulation produced the best results in terms of TTF performance enhancement. 

### 4.1. tDCS Effects on Maximal Voluntary Contraction

It is well established that maximal strength capabilities depends on both muscular and neural resources. Regarding the neural factors limiting maximal force production it is well accepted that motor unit (MU) recruitment strategies play a key role [40]. In this sense, it has been demonstrated that both MU recruitment and synchronization can be modulated through the application of anodal tDCS [41,42]. Therefore, it could be expected that this neuromodulatory technique may elicit some improvements in the MVC, as previously suggested [12,14,22]. However, our analysis indicated small effect of anodal tDCS on maximal strength capabilities. Indeed, only 3 of 13 studies have reported a significant positive effect of anodal tDCS on the maximal voluntary force [12,14,22]. Tanaka et al. [14] reported a significant increase in the dominant leg pinch force without changes either in the non-dominant leg or in the hand pinch force strength after anodal tDCS of the ipsilateral M1. Similar results were found by Vargas et al. [22], who revealed that 20 min of anodal tDCS over the M1 induced an increase in the maximal voluntary contraction of the dominant quadriceps that lasted up to 60 min (average increase of 7.5%). However, no effect was present either in the sham condition or in the non-stimulated limb during the active condition [22]. Nevertheless, because of the electrode size (i.e., 35 cm^2^), the effects of the tDCS would possibly influence the adjacent contralateral leg motor cortex representation and therefore, this should have led to an increased non-dominant leg force. Furthermore, Tanaka et al. [14] used large pad electrodes (35 cm^2^) for stimulating left leg primary motor cortex. However, this electrode size would also stimulate hand motor cortex, which is located only 4 cm away from the vertex, but conversely, they did not reported changes in the right hand pinch force [14]. Therefore, those studies that found positive effects have major inconsistencies regarding the explanation of the observed effect. Based on the observations mentioned above, more studies are needed to further explore the effects of anodal tDCS on maximal voluntary contraction.

### 4.2. The Effect of Anodal tDCS on Time to Task Failure

The present meta-analysis demonstrated a moderate significant effect of anodal tDCS on the Time to Task Failure. 10 of the 18 interventions analyzed have reported an enhanced endurance performance after application of anodal tDCS. The exact mechanisms by which tDCS improves TTF are still unknown. It has been suggested that tDCS likely facilitates the M1 by increasing its output during exercise and possibly reducing supraspinal fatigue [15,17]. In addition anodal tDCS could reduce the Rate of Perceived Exertion (RPE), which might explain the improvement in performance [18,19,20,27]. Thus, if M1 excitability is increased following tDCS administration, it needs to receive less input to generate the amount of output required to recruit the muscle, hence, a lower RPE for a given force or power should be expected [18]. However, up to date, there are some inconsistencies regarding these hypotheses because some studies reported increases in TTF after tDCS application without changes in RPE [30] or changes in M1 excitability [27]. 

Additionally, there are several factors that could influence the results obtained in this meta-analysis such as the type of task performed, the stimulation region, as well as the duration of stimulation. Therefore, we have performed some sub-analysis in order to detect some potential mediators that can influence the observed effects of anodal tDCS on the TTF. 

### 4.3. Stimulation Region

Primary motor cortex (M1) is assumed to control the motor drive that is necessary to activate the motor units and thus, it is commonly considered as a key determinant in endurance tasks [43]. In this regard, the results of different studies analyzed in the present review hold the use of M1 stimulation for improving TTF [15,17,20,29]. Angius et al. [18,20] and Cogiamanian et al. [15] have proposed that increasing the excitability of motor and premotor cortical areas, such as M1 and premotor cortex, may have an impact on RPE (i.e., reduced perception of effort), which would lead to the maintenance of a given power output for a longer time (i.e., increased TTF). But also, there is evidence regarding the role of other cortical regions in the regulation of endurance exercise [44]. For example, a functional magnetic resonance imaging (fMRI) study indicated steady increases in brain activity in the sensorimotor cortex, prefrontal cortex (PFC), cingulate gyrus, supplementary motor area, and cerebellum during sustained contractions [45]. At the top of the motor hierarchy [46], the PFC would be particularly important in the regulation of exercise by modulating the motor drive following integration of both cognitive and peripheral information [44]. Specifically, it may have a motivational function, by inhibiting peripheral fatigue cues signalling the urge to stop, therefore allowing the maintenance of a goal-directed task [16]. In this regard, our data indicated that the magnitude of the effect produced by both M1 and PFC stimulation was moderate (ES = 0.28 versus ES = 0.20, respectively). Therefore, further studies should elucidate, from a mechanistic point of view, the cerebral region which should be targeted to produce the better effects on motor performance, and thus, providing a rationale for using this neuromodulatory technique in the field of sport performance. 

### 4.4. Duration of Stimulation

The current analysis revealed higher effect sizes when stimulating the cerebral cortex for more than 10 min (i.e., 15–20 min) when compared to shorter stimulation durations (i.e., 10 min.; ES = 0.31 versus ES = 0.17, respectively). Stimulation duration has been shown to modulate the length of time before cortical excitability returns to baseline levels post-stimulation [3]. For example, the after-effects of 9 min tDCS lasted up to 30 min, whereas stimulating for 13 min increased this time to 90 min [3]. Therefore, to ensure that the expected effect is long enough to influence endurance performance, it should be recommended to use stimulation duration longer than 10 min.

### 4.5. Whole-Body Exercise Versus Uniarticular-Based Exercise

Our analysis revealed higher effect size (ES = 0.41) for those studies that used whole body exercises (i.e., cycling TTF) when compared to the effect observed when using uniarticular tasks to failure (ES = 0.15). It is known that task selection influences the fatigue [47,48,49], and therefore, the response of the tDCS over the cortex or downstream the corticospinal tract could be also influenced by the type of task. It has been suggested that afferent feedback play a role in the modulation of perception of effort [50,51] and it has a big impact on performance in those tasks requiring sustained submaximal efforts until volitional failure [51]. Therefore, those exercises involving large muscle groups such as locomotor lower limb musculature (i.e., running or cycling), and thus, higher amount of afferent feedback signalling [52], could be greatly influenced by lowering RPE throughout application of anodal tDCS.

## 5. Conclusions

This meta-analysis revealed that anodal tDCS leads to a small improvement in maximal force production, but it seems to produce a moderate positive effect on TTF. Furthermore, stimulation for more than 10 min induces larger ES than 10 min only. Additionally, we found larger ES when anodal tDCS was applied prior to a task involving whole body movements (i.e., cycling) in comparison to the effect observed when using uniarticular tasks to failure. Stimulation of either M1 or prefrontal areas produces moderate effects on TTF (SMD = 0.28 and SMD = 0.20, respectively). However, these conclusions should be taken with caution because the overall effect on TTF is moderate (SMD = 0.26), and any of the sub-analysis showed statistically significant differences between sub-groups (i.e., full-body versus uniarticular; 10 min. versus >10 min.; M1 versus PFC). Furthermore, it seems that tDCS effects on TTF are mainly driven by results obtained for the group that received full-time stimulation in the Williams et al. [17] study (ES = 1.41; 95% CI = 0.28, 2.55). It is also evident that most of the studies analyzed here are underpowered due to small sample sizes. This is also of relevance taking into account the high inter-individual variability in response to tDCS [53]. Our analysis revealed that a sample size of 165 subjects is needed to observe statistical significant effects of anodal tDCS on TTF with a minimum power of 0.8. Therefore, future studies should increase the number of subjects included in their analysis. Another critical issue is the lack of studies using neurophysiological measurements to further understands the neural mechanisms contributing to the performance enhancement by tDCS [18].

In summary, the current meta-analysis indicated that anodal tDCS lead to a small to moderate positive effect on TTF and maximal force production. However, the small sample sizes of the studies included in the analysis and the inconsistency of outcomes make questionable the use of this technique to improve the aforementioned parameters.

## Figures and Tables

**Table 1:** Acute effects of transcranial direct current stimulation (tDCS) on maximal voluntary contraction (*n* = 12).

Study	Sample	tDCS Intensity	Polarity	Stimulation Electrode	Reference Electrode	Duration (Minutes)	Task	Main Outcome (MVC in N, N·m, N/kg)
Tanaka et al. [14]	10 (8 M, 2 F); 27.5 ± 7.5 years	tDCS 2 mA	A/S	M1	Orbital	10	MVC of lower leg pinch force	A: 31.2 ± 8.9 S: 29.5 ± 7.5
Kan et al. [13]	15 (15 M) 27.7 ± 8.4 years	tDCS 2 mA	A/S	M1	Shoulder	10	MVC of elbow flexors	S: 62.2 ± 11.1 A: 62.0 ± 11.2
Montenegro et al. [25]	14 (14 M) 26 ± 4 years	tDCS 2 mA	A/S	M1	Supraorbital	20	MVC of knee extensors	A: 147 ± 29.6 S: 152.1 ± 26
Sales et al. [39]	19 (19 M) 25.2 ± 4 years	tDCS 2 mA	A/S	Temporal lobe	Supraorbital	20	MVC of knee extensors	A: 259 ± 46 S: 233 ± 32
Washabaugh et al. [37]	22 (15 M, 7 F) 22.8 ± 5.7 years	tDCS 2 mA	A/S	M1	Supraorbital	12	MVC of knee extensors	A: 173.1 ± 18.8 S: 176.0 ± 22.9
Angius et al. [27]	9 (9 M) 23 ± 2 years	tDCS 2 mA	A-C/A-E/S	M1	A-E: Shoulder A-C: Right DLPFC	10	MVC of knee extensors	A-E: 250.7 ± 59.7 A-C: 243.5 ± 48.7 S: 234.0 ± 29.8
Frazer et al. [21]	14 (6 M, 8 F) 18–35 years	tDCS 2mA	A/S	M1	Supraorbital	20	MVC of wrist extensors	A: 165 ± 88.1 S: 152.7 ± 77.3
Hazime et al. [12]	8 (8 F) 19.7 ± 2.6 years	tDCS 2 mA	A/S	M1	Supraorbital	20	MVC of external shoulder rotators	A: 1.1 ± 0.2 S: 0.9 ± 0.1
Flood et al. [24]	12 (12 M) 24.4 ± 3.9 years	tDCS 2 mA	A/S	M1	4 cathodes around the anode	20	MVC of knee extensors	A: 236.3 ± 66.5 S: 239.6 ± 67.5
Vargas et al. [22]	20 (20 F) 16.1 ± 0.9 years	tDCS 2 mA	A/S	M1	Supraorbital	20	MVC of knee extensors	A: 10.1 ± 1.5 S: 9.1 ± 1.6
Oki et al. [38]	11 (4 M, 7 W) 85.8 ± 4.3 years	tDCS 1.5 mA	A/S	M1	Supraorbital	20	MVC of elbow flexors	A: 26.9 ± 14.3 S: 27.6 ± 14.3
Angius et al. [20]	12 (8 M, 4 F) 24 ± 5 years	tDCS 2 mA	A/S	M1	Shoulders	10	MVC of knee extensors	A: 214.9 ± 56.4 S: 204.6 ± 60.9

M1: primary motor cortex; DLPFC: dorsolateral prefrontal cortex; PFC: prefrontal cortex; tDCS: transcranial direct current stimulation; TTF: time to task failure; MVC: maximal voluntary contraction; TTE: time to exhaustion; mA: milliamps; M: male; F: female; A: anodal; S: sham; A-C: anodal cephalic; A-E: anodal extracephalic.

**Table 2:** Acute effects of TDCS on TTF (*n* = 15).

Study	Sample	NIBS	Polarity	Stimulation Electrode	Reference Electrode	Duration (Minutes)	Task	Main Outcome (Seconds)
Cogiamanian et al. [15]	24 (10 M, 14 F) 24.3 years	tDCS 1.5 mA	A/S	M1	Shoulder	10	TTF at 35% of MVC of elbow flexors	A: 205.2 ± 24.9 S: 136.1 ± 14.9
Williams et al. [17]	18 (9 M, 9 F) 25 ± 6 years	tDCS 1.5 mA	A/S	M1	Orbitofrontal cortex	20	TTF at 20% MVC of elbow flexors	Full-time stimulation group: A: 988.8 ± 172.2 S: 787.8 ± 80.4 Part-time stimulation group: A: 2000.4 ± 1047.6 S: 2053.8 ± 1076.0
Muthalib et al. [33]	15 (15 M) 27.7 ± 8.4 years	tDCS 2 mA	A/S	M1	Shoulder	10	TTF at 30% of MVC of elbows flexors	A: 333 ± 119 S: 353 ± 146
Kan et al. [13]	15 (15 M) 27.7 ± 8.4 years	tDCS 2 mA	A/S	M1	Shoulder	10	TTF at 30% of MVC of elbow flexors	S: 354.5 ± 144.8 A1: 328.8 ± 122.4
Vitor-Costa et al. [30]	11 (11 M) 26 ± 4 years	tDCS 2 mA	A/S	M1	Occipital protuberance (inion)	13	TTE cycling at 80% of Pmax	A: 487.3 ± 196.6 S: 404.2 ± 136.4
Angius et al. [31]	9 (9 M) 23 ± 4 years	tDCS 2 mA	A/S/C	M1	DLPFC	10	TTE cycling at 70% of Pmax	A: 994.8 ± 509,4 S: 880.8 ± 517.2
Okano et al. [19]	10 (10 M) 33 ± 9 years	tDCS 2 mA	A/S	Left Temporal Cortex	Supraorbital	20	Maximal incremental cycling test	A: 751.4 ± 71.5 S: 723.7 ± 45.0
Angius et al. [27]	9 (9 M) 23 ± 2 years	tDCS 2 mA	A-C/A-E/S	M1	A-E: Shoulder A-C: right DLPFC	10	TTF at 20% of MVC of knee extensors	A-E: 219 ± 136 A-C: 191 ± 124 S: 173 ± 114
Barwood et al. [32]	8 (8 M) 21 ± 1 years	tDCS 2 mA	A/S	PFC	Supraorbital	20	TTE cycling at 70% of Pmax	A: 237 ± 362 S: 314 ± 334
Abdelmoula et al. [26]	11 (8 M, 3 F) 25 ± 1.8 years	tDCS 1.5 mA	A/S	M1	Shoulder	10	TTF at 35% of MVC of elbow flexors	A: 162.4 ± 52.8 S: 148.6 ± 42.7
Oki et al. [29]	13 (5 M, 8 F) 68.3 ± 2 years	tDCS 1.5 mA	A/S	M1	Orbitofrontal cortex	20	TTF at 30% of MVC of elbow flexors	A: 1014 ± 132 S: 882 ± 108
Radel et al. [16]	22 (13 M, 9 F) 21.36 ± 0.43 years	tDCS 2 mA	A/S	PFC and M1	4 cathodes around anode	10	TTF at 35% of MVC of elbow flexors	A-PFC: 251.2 ± 245.5 A-M1: 267.6 ± 149.6 S: 247.3 ± 168.1
Flood et al. [24]	12 (12 M) 24.42 ± 3.85 years	tDCS 2 mA	A/S	M1	4 cathodes around anode	20	TTF at 30% of MVC of non dominant knee extensors	A: 93.1 ± 33.7 S: 100.3 ± 44.3
Lattari et al. [28]	11 (11 F) 24.0 ± 2.2 years	tDCS 2 mA	A/S	DLPFC	Orbitofrontal cortex	20	TTE cycling at 100% of Pmax	A: 199.5 ± 97.2 S: 137.1 ± 73.1
Angius et al. [20]	12 (8 M, 4 F) 24 ± 5 years	tDCS 2 mA	A/S	M1	Shoulders	10	TTE cycling at 70% of Pmax	A: 795 ± 260.4 S: 645.6 ± 181.8

M1: primary motor cortex; DLPFC: dorsolateral prefrontal cortex; PFC: prefrontal cortex; tDCS: transcranial direct current stimulation; TTF: time to task failure; MVC: maximal voluntary contraction; TTE: time to exhaustion; Pmax: peak power output of an incremental cycling test; mA: milliamps; M: male; F: female; A: anodal; S: sham; A-C: anodal cephalic; A-E: anodal extracephalic.

## Supplementary Materials

The following are available online at https://www.mdpi.com/2077-0383/8/4/536/s1, Figure S1: Funnel plot for MVC (A) and TTF (B) studies; Table S1: PEDro ratings of the qualitative assessment.
