# Supplementary material for: Short-Term Effects of Anodal Transcranial Direct Current Stimulation on Endurance and Maximal Force Production: A Systematic Review and Meta-Analysis
Source: J Clin Med. 2019 Apr 18;8(4):536. doi: 10.3390/jcm8040536 (PMC6518246; doi:10.3390/jcm8040536)
Supplement: Supplementary file 1 [file jcm-08-00536-s001.pdf]

Table S1. PEDro ratings of the qualitative assessment.

		Abdelmoula et al. (2016)	Angius et al. (2015)	Angius et al. (2016)	Angius et al. (2018)	Barwood et al. (2016)	Cogiamanian et al. (2007)	Flood et al. (2017)	Frazer et al. (2016)	Hazime et al. (2017)	Kan et al. (2013)	Lattari et al. (2018)	Montenegro et al. (2015)	Muthalib et al. (2013)	Okano et al. (2015)	Oki et al. (2016)	Oki et al. (2017)	Radel et al. (2017)	Sales et al. (2016)	Tanaka et al. (2009)	Vargas et al. (2017)	Vitor-Costa et al. (2015)	Washabaugh et al. (2016)	Williams et al. (2013)
1	Eligibility criteria were specified	Y	N	N	N	N	N	Y	N	N	N	N	Y	N	N	Y	Y	Y	Y	N	Y	Y	Y	Y
2	Subjects were randomly allocated to groups	Y	Y	Y	Y	Y	Y	Y	Y	Y	Y	Y	Y	Y	Y	Y	Y	Y	Y	Y	Y	Y	Y	Y
3	Allocation was concealed	N	N	N	N	Y	Y	N	Y	Y	N	Y	N	N	N	N	N	N	N	N	Y	N	Y	N
4	The groups were similar at baseline regarding the most important prognostic indicators	Y	Y	Y	Y	Y	Y	Y	Y	Y	Y	Y	Y	N	Y	Y	Y	Y	Y	Y	Y	Y	Y	Y
5	There was blinding of all subjects	Y	Y	Y	Y	Y	Y	Y	Y	Y	Y	Y	Y	N	Y	Y	Y	Y	Y	N	Y	Y	N	Y
6	There was blinding of all therapists who administered the therapy	N	N	N	N	N	N	N	Y	N	N	N	N	N	N	N	N	Y	N	N	N	N	N	N
7	There was blinding of all assessors who measured at least one key outcome	N	N	N	N	N	N	N	N	Y	N	Y	Y	N	Y	Y	Y	Y	Y	N	Y	N	N	N
8	Measures of at least one key outcome were obtained from more than 85% of the subjects initially allocated to groups	Y	Y	Y	Y	Y	Y	Y	Y	Y	Y	Y	Y	Y	Y	Y	Y	Y	Y	Y	Y	Y	Y	Y
9	All subjects for whom outcome measures were available received the treatment or control condition as allocated or, where this was not the case, data for at least one key outcome was analysed by "intention to treat"	Y	Y	Y	Y	Y	Y	Y	Y	Y	Y	Y	Y	Y	Y	Y	Y	Y	Y	Y	Y	Y	Y	Y
10	The results of between-group statistical comparisons are reported for at least one key outcome	Y	Y	Y	Y	Y	Y	Y	Y	Y	Y	Y	Y	Y	Y	Y	Y	Y	Y	Y	Y	Y	Y	Y
11	The study provides both point measures and measures of variability for at least one key outcome	N	N	N	Y	N	N	Y	Y	N	Y	N	N	Y	N	Y	Y	Y	N	Y	Y	N	Y	Y
	Total	6	7	6	7	7	7	7	9	8	7	8	7	5	7	8	8	9	7	6	9	6	7	7

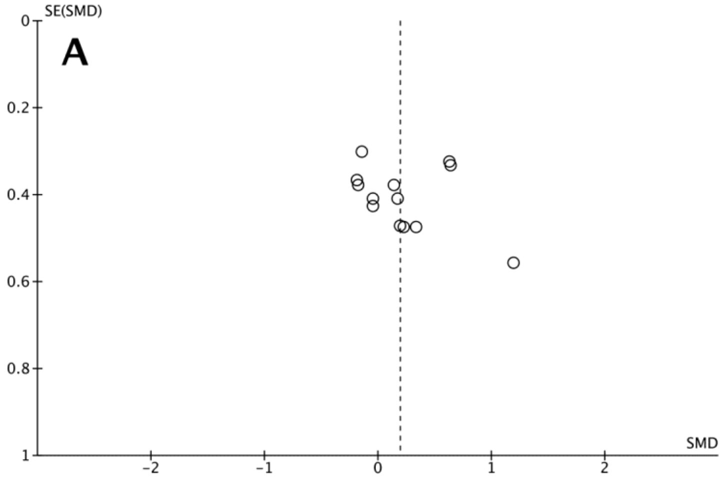

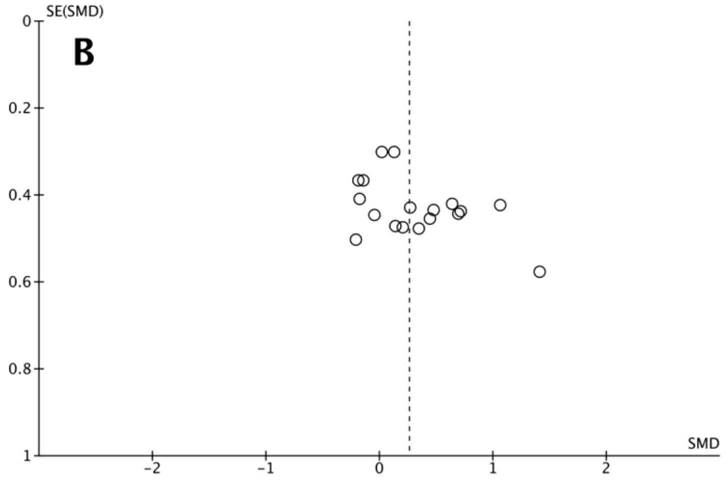

Figure S**1.** Funnel plot for MVC (A) and TTF (B) studies.
